# Comparison between diagnosis and treatment of community-acquired pneumonia in children in various medical centres across Europe with the United States, United Kingdom and the World Health Organization guidelines

**DOI:** 10.1186/s41479-016-0005-y

**Published:** 2016-05-02

**Authors:** Vytautas Usonis, Rimvydas Ivaskevicius, Javier Diez-Domingo, Susanna Esposito, Oana G. Falup-Pecurariu, Adam Finn, Fernanda Rodrigues, Vana Spoulou, George A. Syrogiannopoulos, David Greenberg, CAP-PRI Working Group

**Affiliations:** 1 Clinic of Children’s Diseases, Vilnius University, Vilnius, Lithuania; 2 FISABIO-Public Health, (CSISP), Valencia, Spain; 3 Department of Maternal and Paediatric Sciences, Università degli Studi di Milano Fondazione IRCCS Ca’ Granda Ospedale Maggiore Policlinico, Milano, Italy; 4 University Children’s Hospital, Transylvania University, Brasov, Romania; 5 Bristol Children’s Vaccine Centre, School of Clinical Sciences, University of Bristol, Bristol, UK; 6 Infectious Diseases Unit & Emergency Service, Hospital Pediátrico, Centro Hospitalar e Universitário de Coimbra, Coimbra, Portugal; 7 First Department of Paediatrics, Agia Sophia Children’s Hospital, Athens, Greece; 8 Department of Paediatrics, General University Hospital of Larissa, Larissa, Greece; 9 School of Health Sciences, Faculty of Medicine, University of Thessaly, Larissa, Greece; 10 The Paediatric Infectious Disease Unit, Soroka University Medical Center, Beer-Sheva, Israel; 11 Faculty of Health-Sciences, Ben-Gurion University of the Negev, Beer-Sheva, Israel

**Keywords:** Community-acquired pneumonia, Guidelines, Community-acquired pneumonia diagnosis, Community-acquired pneumonia treatment, Antibacterial treatment

## Abstract

**Background:**

The aim of this study was to review the current status and usage of guidelines in the diagnosis and treatment of community-acquired pneumonia (CAP) in European countries and to compare to established guidelines in the United States (US), United Kingdom (UK), and the World Health Organization (WHO).

**Methods:**

A questionnaire was developed and distributed by the Community-Acquired Pneumonia Paediatric Research Initiative (CAP-PRI) working group and distributed to medical centres across Europe.

**Results:**

Out of 19 European centres, 6 (31.6 %) used WHO guidelines (3 in combination with other guidelines), 5 (26.3 %) used national guidelines, and 5 (26.3 %) used local guidelines. Chest radiograph and complete blood count were the most common diagnostic examinations, while evaluation of clinical symptoms and laboratory tests varied significantly. Tachypnoea and chest recession were considered criteria for diagnosis in all three guidelines. In US and UK guidelines blood cultures, atypical bacterial and viral detection tests were recommended. In European centres in outpatient settings, amoxicillin was used in 16 (84 %) centers, clarithromycin in 9 (37 %) centers and azithromycin in 7 (47 %) centers, whereas in hospital settings antibiotic treatment varied widely. Amoxicillin is recommended as the first drug of choice for outpatient treatment in all guidelines.

**Conclusions:**

Although local variations in clinical criteria, laboratory tests, and antibiotic resistance rates may necessitate some differences in standard empirical antibiotic regimens, there is considerable scope for standardisation across European centres for the diagnosis and treatment of CAP.

**Electronic supplementary material:**

The online version of this article (doi:10.1186/s41479-016-0005-y) contains supplementary material, which is available to authorized users.

## Background

Community-acquired pneumonia (CAP) is a common cause of childhood morbidity and mortality worldwide [1]. In North America and Europe, the annual incidence of CAP was estimated to be approximately 34–40 cases per 1000 in children under 5 years of age [2], prior to widespread use of pneumococcal conjugate vaccines (PCVs). There are several major difficulties in the diagnosis and treatment of CAP in children. First, the case definitions for pneumonia are not standardised and can vary by region and even by hospital. The diagnosis of pneumonia is usually based on a patient’s history, clinical signs, and laboratory test results, such as complete blood count (CBC) and chest radiograph [3]. The World Health Organization (WHO) has defined pneumonia entirely based on clinical findings obtained by simple observations [4, 5], although the specificity and sensitivity of clinical signs for identifying pneumonia are relatively low [5–7]. Second, determining the aetiologic agent of pneumonia in children is problematic because there is no accepted "gold standard". Blood cultures have low sensitivity and lung puncture is not performed routinely [3]. The distribution of causative agents of CAP varies with age, and respiratory virus infections are more common in early childhood [1, 8]. *Streptococcus pneumoniae* is a major bacterial cause in all age groups after the neonatal period and is often associated with complications [9, 10]. Furthermore, co-infection with both bacteria and virus can be found mainly in the first years of life in approximately 30 % of all cases [1, 8–10]. Third, antibiotic resistance patterns and vaccine use differ across Europe, making it difficult to adopt one guideline for the whole continent [11, 12]. Recently, new guidelines from the United States (US) [1] and the United Kingdom (UK) [13] were published with specific recommendations regarding the diagnosis and treatment of CAP in children.

The aims of this study were to determine whether local guidelines for the diagnosis and treatment of CAP in children are available in European countries; to compare these guidelines and practices with regard to diagnosis and treatment; and to compare these local guidelines with published guidelines from the Pediatric Infectious Diseases Society and the Infectious Diseases Society of America (PIDS-IDSA) [13], the British Thoracic Society (BTS) [1], and the WHO [15].

## Methods

### Study design, setting and participants

This was a snapshot prospective study based on a questionnaire sent to paediatricians and paediatric infectious disease specialists across Europe from October 2010 to December 2012. The study was conducted by the Community-Acquired Pneumonia Paediatric Research Initiative (CAP-PRI) working group. CAP-PRI is a consortium convened under the auspices of the European Society of Paediatric Infectious Diseases (ESPID), which includes eight countries across Europe (Greece, Israel, Italy, Lithuania, Portugal, Romania, Spain, and UK). In each country at least one major paediatric hospital or service is included in the consortium.

The questionnaire was emailed by the Lithuanian group to 22 medical centres in 20 European countries. The selection of the centres was based on the CAP-PRI working group and other centres across Europe that responded to an email request sent to their center and were interested in participating in the study.

### Development of the questionnaire

An original questionnaire was developed by the members of CAP-PRI (Additional file 1); it was based on previous published guidelines [1, 13, 14] and previous publications on the diagnosis and treatment of CAP in children [9, 15]. It consisted of 14 questions covering the following 5 sections: (i) a general section describing the use of guidelines at the centres including local or international recommendations; (ii) use of clinical criteria for the diagnosis of pneumonia (cough, fever, tachypnoea, intercostal/subcostal/suprasternal retractions, nasal flaring, crackles, decreased breath sounds, hypoxaemia/oxygen saturation <95 %, dehydration, abdominal pain, consolidation in chest radiograph); (iii) use of diagnostic criteria such as chest radiograph and specific laboratory tests (CBC, C-reactive protein [CRP], erythrocyte sedimentation rate [ESR], procalcitonin [PCT], serum electrolytes, blood culture, nasopharyngeal culture, sputum culture, rapid diagnostic tests [respiratory syncytial virus, adenovirus, human metapneumovirus], serology) for the diagnosis of pneumonia; (iv) criteria for hospitalisation such as age, hypoxaemia, moderate to severe respiratory distress, underlying disease, dehydration, inability to feed, treatment failure, and inadequate family supervision (v) antibiotic treatment, including local recommendations for first and second lines of treatment and the variations by age and location (both outpatient and hospitalised inpatient).

The questionnaire was drafted in Lithuania and was then circulated among the CAP-PRI group members and modified according to the suggestions received. Questionnaires were not validated as there were no subjective questions, and we requested the hospitals to respond based on their local guidelines and policy rather than the opinions of the individual completing the questionnaire.

### Data collection

Questionnaire data were submitted to and recorded by the Lithuanian group using Access Microsoft Office 2010 software (US). Every effort was made to re-contact centres for information if there were any incomplete responses or inconsistencies. Data was then passed to the FISABIO-Public Health, (CSISP) group in Valencia, Spain, for validation and statistical analysis.

### Statistics

Statistical analysis was conducted by the Spanish group using SPSS 18.0 software (SPSS Inc., US). Contingency table analysis measured the association among centres using the two tails *χ*2 tests or Fisher’s exact test. More than one answer was possible in most of the questions.

## Results

**Fig. 1:**
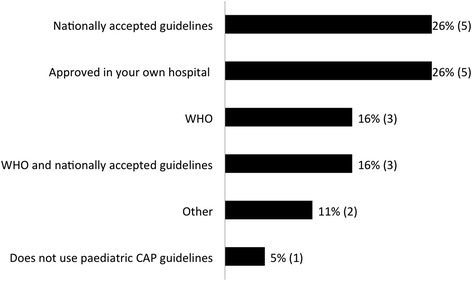
International, national, and local guidelines usage for diagnosis and treatment of community-acquired pneumonia in children in European medical centres. WHO, World Health Organization; CAP, community-acquired pneumonia

Nineteen (86.3 %) public or teaching university hospital medical centres returned the questionnaires from 16 (80.0 %) countries: Lithuania, Latvia, Estonia, Belarus, Israel, Spain (*n* = 2), Italy, Romania, Greece (*n* = 3), Croatia, Portugal, Bulgaria, Slovenia, Ukraine, Slovak Republic, and UK. All 19 centres (16 responding countries) reported to have locally available CAP guidelines. Three out of 19 (15.8 %) responding centres use WHO guidelines, 5 (26.3 %) centres use national guidelines, 5 (26.3 %) use guidelines approved by their hospital, 3 (15.8 %) centres use WHO and national guidelines, 2 (10.5 %) use other guidelines and 1 (5.3 %) centre did not use paediatric CAP guidelines (Fig. 1).

### Criteria for the diagnosis of CAP

**Fig. 2:**
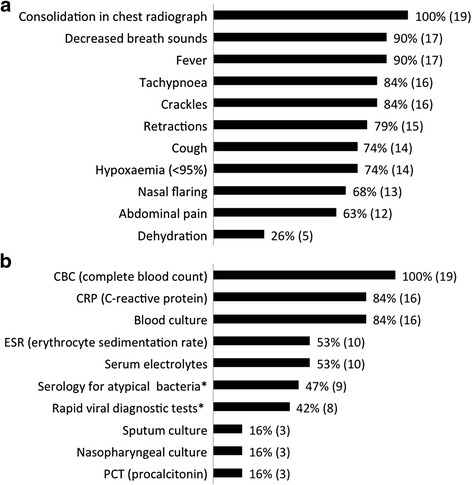
Percentage of European medical centres that use the specified clinical and laboratory diagnostic parameters for community-acquired pneumonia in children in European medical centres. **a** Clinical parameters **b** Laboratory tests. *Includes *Mycoplasma* and *Chlamydia* for serology and respiratory syncytial virus, adenovirus, and influenza virus for viral diagnostic tests

Consolidation in chest radiograph was the diagnostic criteria for CAP at all centres, and for 2 centres it was the only requirement (Fig. 2a). At another centre, the presence of fever and decreased air entry was also required in addition to the chest radiograph finding. The most commonly used signs were fever (17 [89.5 %]), decreased breath sounds (17 [89.5 %]), tachypnoea (16 [84.2 %]), and crackles (16 [84.2 %]). While these clinical signs along with chest radiographs (19 [100 %]) were the major diagnostic criteria for CAP in centres across Europe, others were widely used: intercostal and subcostal retractions (15 [79.0 %]), cough and oxygen saturation <95 % (14 [73.7 %]), and nasal flaring (13 [68.4 %]) (Fig. 2a). Abdominal pain and dehydration as diagnostic criteria were used in 62.3 % (*n* = 12) and 26.3 % (*n* = 5) of centres, respectively, that completed the questionnaire.

Rapid respiratory rate (above the upper normal limit for age) and chest recession were considered criteria for diagnosis in all three guidelines (PIDS-IDSA [13], BTS [1], and WHO [14]) (Table 1). WHO and PIDS-IDSA guidelines also included grunting, nasal flaring, and oxygen saturation of less than 90 %. Additionally, PIDS-IDSA guidelines considered altered mental status as a criterion for diagnosis and WHO considered general danger signs such as lethargy as clinical signs and symptoms to determine CAP. In the BTS guidelines, temperature above 38.5 °C was a criterion for bacterial pneumonia, while in other guidelines temperature was not used as a criterion. Only WHO guidelines determined severity of CAP based on signs and symptoms.

**Table 1:** Comparison of clinical signs and symptoms to determine community-acquired pneumonia (CAP) severity among different European medical centres compared with the United States [13], United Kingdom [1] and World Health Organization [14] guidelines

Sign or symptom	Guidelines			European study^a^
	PIDS-IDSA	BTS^b^	WHO	
Tachypnoea	✓^c^	✓	✓^c^	✓
Chest recession/indrawing/retractions	✓	✓	✓	✓
Nasal flaring	✓			✓
Cough			✓^d^	✓
Grunting	✓		✓^d^	
Apnoea	✓			
Fever		✓		✓
Difficulty breathing/respiratory distress	✓		✓^d^	✓
Low oxygen saturation	✓ (<90 %)	✓ (<92 %)	✓^d^ (<90 %)	✓ (<95 %)
Abdominal pain				✓
General danger signs (inability to drink, vomiting, lethargy, convulsions)			✓^d^	
Altered mental status	✓			
Cyanosis			✓^d^	
Auscultation revealing absent breath sounds with a dull percussion note or crackles		✓	✓	✓

*PIDS-IDSA* Pediatric Infectious Diseases Society and the Infectious Diseases Society of America, *BTS* British Thoracic Society, *WHO* World Health Organization

^a^Indications for hospitalisation, only when >50 % of medical centres reported using the parameter

^b^Recommendations for bacterial pneumonia

^c^Respiratory rate adjusted by age

^d^Symptom of severe CAP

### Indication to obtain a chest radiograph

In all European centres chest radiograph was a criterion for the diagnosis of pneumonia when performed. All centres recommended chest radiograph for children admitted to the hospital emergency room or hospitalised children. While 13 (68.4 %) centres performed a chest radiograph when pneumonia was suspected, 4 (21.1 %) centres requested it only when hospitalisation was required, and 2 (10.5 %) centres requested it when treatment failed or when a complication of pneumonia was suspected. Eleven (57.9 %) of the centres had various indications for chest radiograph, including children less than 5 years of age with fever without focus.

Although it varied from center to center whether WHO guidelines or other guidelines for the diagnosis of CAP in children were used, no significant variations in the indication for chest radiograph were found between the groups. However, indication for chest radiograph was related to whether the guidelines were applied in a hospital or in the community. Thus, in the case of the 6 medical centres applying the guidelines in an outpatient setting, chest radiograph was less frequently indicated when CAP was suspected (33 % vs 85 %; *p* < 0.05), or when findings were ambiguous (0 % vs 50 %; *p* < 0.05). Although not recommended routinely by the guidelines used in European centres, some responding centres performed follow-up chest radiograph when there was an indication. The most common indications were pleural effusion requiring drainage in 17 (89.5 %) centres, persistence of clinical symptoms after treatment with an adequate antibacterial drug in 16 (84.2 %) centres, and lung abscess in 16 (84.2 %) centres.

In all guidelines chest radiograph was not a necessary condition for the diagnosis of CAP in children in outpatient settings. For hospitalised patients all guidelines stated that chest radiograph should be performed. The PIDS-IDSA guidelines state that “chest radiographs, postero-anterior and lateral, should be obtained,” [13] while the BTS guidelines state that “a lateral X-ray should not be performed routinely” [1]. The WHO guidelines [15] recommends a chest radiograph when possible in all severe cases of pneumonia. Follow-up radiographs were recommended in the PIDS-IDSA and BTS guidelines only in cases of complicated pneumonia (i.e., pleuropneumonia or lung abscess) or in non-responsive cases.

### Laboratory diagnostics

All centres performed a CBC while 16 (84.2 %) tested for different inflammatory markers such as CRP in hospitalised patients (Fig. 2b). Other diagnostic methods frequently used were blood culture (16 [84.2 %]), serology for various respiratory pathogens (9 [47.4 %]), and rapid viral diagnostic tests (8 [42.1 %]).

None of the guidelines recommend routine laboratory tests for outpatients. For hospitalised patients, CBC, ESR, PCT and CRP are recommended by the PIDS-IDSA guidelines but not by the BTS guidelines (Table 2), which recommend performing these tests only for children with severe or complicated CAP. In severe cases, all guidelines (except the WHO guidelines) recommended CBC, blood cultures, and viral studies (by polymerase chain reaction, antigen detection or serology). Several centres reported using mycoplasma serology as well. Pneumococcal urine antigen test is not recommended by any guidelines for CAP in children.

**Table 2:** Comparison of inpatient diagnostic test indications for community-acquired pneumonia (CAP) across different European medical centres compared with United States [13] and United Kingdom [1] guidelines

Diagnostic test	Guideline		European study
	PIDS-IDSA	BTS	
Chest radiograph	Yes	Yes	Yes
Complete blood count	Yes^a^	No	Yes
Acute phase reactants (CRP, serum PCT, ESR)	Yes^a,b^	No	Yes
Sputum samples for bacteria	Yes	Not specified	Yes^c^
Tests for *Mycoplasma*, *Chlamydia* ^d^	Yes	Yes	Yes^c^
Tests for respiratory viruses^d^	Yes	Yes	Yes^c^
Blood culture	Yes	Yes	Yes
Nasopharyngeal secretions	Not specified	Yes	Yes^c^
Serum electrolytes	Not specified	Not specified	Yes
Not recommended	Urinary antigen detection for pneumococcus Diagnostic testing for *Chlamydophila pneumoniae*	Urinary Antigen detection for pneumococcus Acute phase reactants	
Other	Tracheal aspirates for gram stain and culture	Pleural fluid for microscopy, culture and antigen detection	

With the exception of a chest radiograph, the World Health Organization does not mention use of specific inpatient diagnostic testing and is excluded from the table

*PIDS-IDSA* Pediatric Infectious Diseases Society and the Infectious Diseases Society of America, *BTS* British Thoracic Society, *ESR* erythrocyte sedimentation rate, *CRP* C-reactive protein, *PCT* procalcitonin

^a^Diagnostic test recommended only for those with severe disease

^b^Acute phase reactants cannot be used as the sole determinant to distinguish between viral and bacterial causes of CAP

^c^Tests recommended in <50 % of medical centres

^d^Serology, polymerase chain reaction, culturing and other tests are available but no specific test is recommended

### Criteria for hospitalisation

Recommended criteria for hospitalisation were similar among all of the responders in all centres. The indications for hospitalisations were hypoxaemia (oxygen saturation <92 %, cyanosis), moderate to severe respiratory distress, dehydration, inability to feed, and inability of the family to provide appropriate observation or supervision. In most centres, underlying conditions, outpatient antibiotic treatment failure, or young age (<6 months) were also indications for hospitalisation.

In the PIDS-IDSA guidelines, children and infants with moderate to severe CAP (respiratory distress and hypoxaemia), infants less than 3–6 months with suspected bacterial CAP, or children with CAP caused by a pathogen with increased virulence should be hospitalised. In the BTS guidelines children who have oxygen saturations <92 % or have auscultation revealing absent breath sounds with a dull percussion note should be referred to hospital for assessment and management. In the WHO guidelines, any child that has a cough or difficult breathing with chest indrawing or stridor should urgently be referred to the hospital. In addition, the WHO guidelines add that any child with an oxygen saturation <90 % or central cyanosis should be admitted to the hospital.

### Treatment of CAP

**Fig. 3:**
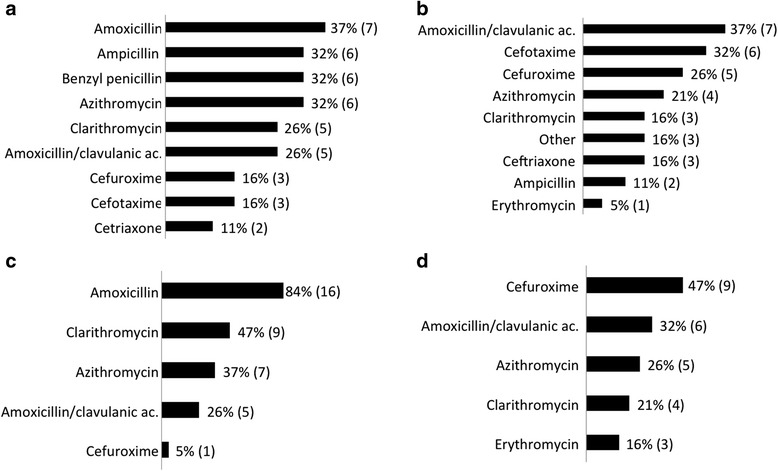
Percentage of European medical centres that use various specified antibiotics for outpatient and inpatient treatment of community acquired pneumonia in children in European medical centres. **a** Inpatient first-line treatment **b** Inpatient second-line treatment **c** Outpatient first-line treatment. **d** Outpatient second-line treatment. Please note that some participating medical centres use more than one antibiotic

Antibacterial treatment for CAP varied across participating centres. In all 19 centres in hospitalised patients, the first-line treatment included either a penicillin or aminopenicillin (Fig. 3a). Amoxicillin was used as a first-line treatment in 7 centres; however, penicillin, ampicillin, or cefuroxime were also recommended as a parenteral treatment. In 11 of 19 (57.9 %) centres, a macrolide was also recommended as a first-line treatment. As a second-line treatment either amoxicillin/clavulanate or a second or third generation cephalosporin was most often recommended for hospitalised children (Fig. 3b). Most (84.2 %) centres used amoxicillin as a first-line antibiotic treatment in outpatients (Fig. 3c). Clarithromycin was also recommended as first-line or an additional treatment in 9 (47.4 %) centres, and azithromycin in 7 (36.8 %) centres. As a second-line antibiotic treatment in outpatients, cefuroxime or amoxicillin/clavulanate were mostly recommended in centres, 9 (47.4 %) and 6 (32 %) respectively (Fig. 3d). In cases of penicillin allergy, macrolides were recommended.

In 17 (89.5 %) centres, treatment was related to age (data not shown). Amoxicillin was usually recommended as the first choice treatment for oral antibiotic therapy in all age groups. In ambulatory children older than 5 years, amoxicillin with or without macrolides was recommended in more than 50 % of the centres for first-line treatment. For hospitalised patients older than 5 years, macrolides either as a single treatment or in combination with benzyl penicillin or cefotaxime were recommended.

Amoxicillin is recommended as the drug of choice for outpatient treatment in all guidelines (Table 3). The WHO guidelines recommend only amoxicillin for less severe pneumonia, ampicillin and gentamicin for more severe inpatient cases. The BTS guidelines recommend oral treatment even in patients with severe pneumonia. When intravenous (IV) treatment was mentioned it included in the first-line antibiotic treatment amoxicillin/clavulanate, cefuroxime and cefotaxime or ceftriaxone. In cases when pneumonia is associated with influenza, co-amoxicillin/clavulanate is recommended. Macrolides are recommended in children older than 5 years by the PIDS-IDSA and BTS guidelines in cases when atypical pneumonia is suspected.

**Table 3:** Comparison of antimicrobial empiric therapy recommendations for children with community-acquired pneumonia across different European medical centres compared with the United States [13], United Kingdom [1] and World Health Organization [14] guidelines

Site of care	Empiric therapy			
	Guideline			European study^a^
	PIDS-IDSA	BTS	WHO	
Outpatient				
First-Line	Amoxicillin	Amoxicillin	Amoxicillin	Amoxicillin Clarithromycin Azithromycin
Second Line	Macrolides^b^ Azithromycin Clarithromycin Erythromycin	Macrolides^c^ Erythromycin Azithromycin Clarithromycin Co-amoxiclav^d^ Cefaclor Ceftriaxone	Not Specified	Cefuroxime Amoxicillin/Clavulanic ac.
Inpatient				
First-line	Ampicillin Penicillin G	Amoxicillin	Ampicillin (or benzylpenicillin) and Gentamicin	Amoxicillin Ampicillin Benzyl penicillin Azithromycin
Second-Line	Cephalosporin^e^ ß-lactam^c^ Vancomycin or Clindomycin^f^	Macrolides^c^ Co-amoxiclav Cefuroxime Cefotaxime Ceftriaxone	Gentamicin Cloxacillin Ceftriaxone	Amoxicillin/Clavulanic ac. Cefotaxime

*PIDS-IDSA* Pediatric Infectious Diseases Society and the Infectious Diseases Society of America, *BTS* British Thoracic Society, *WHO* World Health Organization

^a^Only drugs recommended in >30 % of the medical centres are shown, none of these drugs were recommended in >50 % of medical centres

^b^For atypical pathogens, preferred and alternative agents for specific pathogens are extensively listed in [4]

^c^For children in whom *Mycoplasma pneumoniae* and *Chlamydophila pneumoniae* are significant considerations

^d^For pneumonia associated with influenza

^e^For hospitalised infants and children who are not fully immunised

^f^In addition to ß-lactam therapy if *Staphylococcus aureus* suspected

The second-line treatment recommendations are widely variable and depend on age and immunisation status (PIDS-IDSA guidelines) (Table 3). Macrolides are mostly recommended as a single drug or in combination with ß-lactam antibiotic. For hospitalised patients, ampicillin and penicillin G are recommended by all guidelines. However, for unimmunised patients, third generation cephalosporins are recommended by the PIDS-IDSA guidelines. In addition, it is recommended in the PIDS-IDSA guidelines that vancomycin or clindamycin be used in cases caused by *Staphylococcus aureus* infections and levofloxacin for children who have reached growth maturity, or who cannot tolerate macrolides.

## Discussion and conclusions

This survey reveals the great variety of clinical and laboratory criteria used in the diagnosis and treatment of CAP in children within Europe. Although no standardised guidelines for CAP in children have been established for all of Europe, according to this study European centres do tend to adopt guidelines, most commonly, nationally recognised guidelines, hospital specific guidelines, or guidelines adapted from the WHO. Chest radiograph examination, CBC, and CRP are the most common diagnostic criteria in the European clinics, while evaluation of clinical symptoms and other laboratory tests vary significantly. Antibiotics used for treatment in outpatient settings such as amoxicillin and macrolides were more uniformly used across centres; however, antibiotic treatment varied widely in hospital settings.

Despite the heterogeneity in diagnostic practices seen within European centres, some general similarities exist when comparing local European practices to BTS [1], PIDS-IDSA [13], and WHO [14] guidelines. In the majority of European centres and in the BTS and the PIDS-IDSA guidelines, chest radiograph was always recommended for inpatients when pneumonia was suspected, but was not necessary in an outpatient setting. The WHO recommends performing a chest radiograph, if possible, mainly for identification of complicated pneumonia. Regarding laboratory diagnostics for CAP, European guidelines and practice are most similar to PIDS-IDSA guidelines. Both use laboratory diagnostics such as CBC, acute phase reactants, and blood culture. No diagnostic laboratory tests are indicated in the WHO guidelines. The BTS guidelines are unique in advising against acute phase reactants and do not mention CBC. The BTS guidelines reason that acute phase reactants are not of clinical utility in distinguishing viral from bacterial infections. A distinguishing factor of the PIDS-IDSA guidelines from the European centers and WHO and BTS guidelines regarding diagnosis is that the PIDS-IDSA guidelines emphasise the importance of distinguishing viral from bacterial pneumonia and recommend use of sensitive and specific tests for rapid diagnosis of viral disease. In addition, PIDS-IDSA guidelines strongly recommend use of the influenza virus test since it may decrease both the need for additional diagnostic studies and antibiotic use.

In comparing European center practices to guidelines for outpatient treatment of CAP, it is seen that amoxicillin is consistently used and recommended as the first-line antibiotic therapy. Use of a narrow-spectrum antibiotics such as penicillins or aminopenicillin as a standard of treatment is important since it will decrease future antibiotic resistance rates. Second-line antibiotic usage in European centres is consistent with the PIDS-IDSA and BTS guidelines, suggesting the use of macrolides (although which type of macrolide varies). Although inpatient treatment was widely variable, first-line treatment in European centres was most consistent with PIDS-IDSA guidelines with use of ampicillin as a first-line antibiotic. Additionally, fairly rapid changes in the inpatient management of CAP in the last 15–20 years include a shift from routine IV to oral therapy [1, 8].

PIDS-IDSA guidelines also emphasise the importance of pairing treatment to the specific pathogen causing the pneumonia and extensively list specific antibiotics for atypical pneumonia, while the BTS and WHO guidelines, for the most part, do not distinguish different antibiotics by pathogen. However, the WHO guidelines specify that a child with a confirmed case of staphylococcal pneumonia should be treated with cloxacillin and gentamicin. Pathogen-specific antibiotic treatment in the PIDS-IDSA guidelines probably results directly from the recommendation for laboratory tests which identify the pathogen at diagnosis in an effort to prevent antibiotic resistance. Unlike other guidelines, PIDS-IDSA guidelines also advise more specific antibiotic treatment for older children. For children over the age of 5 years with presumed atypical pneumonia, macrolides can be prescribed in addition to amoxicillin, doxycycline can be prescribed for children over 7 years of age, and levofloxacin can be prescribed for children who have reached growth maturity or who cannot tolerate macrolides. Despite such specific recommendations, compliance with the PIDS-IDSA guidelines remains somewhat low probably due to potential barriers to guideline adherence including lack of guideline awareness, clinician attitudes towards standardisation, lack of agreement with recommendations, and inertia of previous practice [15].

Since there are few randomised studies—especially in the developing world—about duration of antimicrobial treatment, recommendations about scheduled treatment vary between centres and guidelines. Antibiotic treatment is recommended for a duration of 10 days in the PIDS-IDSA guidelines, while the WHO recommends treatment for 5 days and the BTS guidelines do not specify duration of treatment. In all guidelines the dosage of amoxicillin antibiotic was similar (80–90 mg/day). More studies should be performed to elucidate optimal duration of antibiotic treatment for CAP [18].

All guidelines acknowledge that aetiology of pneumonia can be influenced by age. The PIDS-IDSA guidelines state that antimicrobial therapy is not routinely required for preschool-aged children with CAP, because viral pathogens are responsible for the great majority of clinical disease. The BTS guidelines indicate that children under 2 years old with mild symptoms do not usually have bacterial pneumonia and should not be treated with antibiotic. The WHO points out that unless the child has clear signs of moderate to severe pneumonia, the patient is unlikely to have bacterial pneumonia and should not be given antibiotics. Rather, a trial of rapid acting inhaled bronchodilator should be started and only if no improvement is observed, antibiotic treatment can be considered. Various studies show that viral and bacterial infection co-existence is prevalent [16, 17] and thus antibiotics should be considered even in cases with symptoms consistent with viral infections. Viral and bacterial co-infection were not addressed in any of the guidelines and should be addressed in future management recommendations.

During the last 20 years, *Haemophilus influenzae* type b (Hib) and pneumococcal vaccines were implemented in many countries. These two pathogens were responsible for much severe bacterial disease in children [19, 20]. In countries that implemented these vaccines (mainly after the introduction of PCV) a significant reduction in pneumonia rates were reported as well as in antibiotic resistance to pneumococcus [21, 22]. However, only the PIDS-IDSA guidelines make a distinction between antibiotics that should be administered to immunised versus non-immunised children. The guidelines also recommend that children be immunised with vaccines against bacterial pathogens, including *S. pneumoniae*, Hib, and pertussis to prevent CAP. The BTS guidelines acknowledge that vaccination has had a major impact on pneumonia and child mortality worldwide and that PCVs have led to an approximate 30 % decrease in radiologically confirmed pneumonia episodes in young children [1]. The WHO also recommends that all routine childhood immunisation programs include vaccines protecting against influenza virus, measles, pertussis, Hib, and pneumococcus [23]. Despite the rise in the use of vaccines, antibiotic resistance continues to constitute a significant problem. *S. pneumoniae* still has high rates of antibiotic resistance in various regions worldwide including Europe [24, 25, 26].

Although the survey attempts to display the current status of diagnosis and treatment of children with CAP in Europe, it has some limitations. First, most of the participating countries come from southern and eastern Europe, while northern European countries are under-represented. Additionally, only one to two centres were surveyed per country and almost all centres surveyed were large academic centres, and thus the data collected may not accurately reflect guideline use in other paediatric practices and departments. Results are also based on a self-reported questionnaire, and may not accurately reflect the actual diagnostic and treatment methods practiced in the clinic. It is also impossible to know if the PIDS-IDSA, BTS, and WHO guidelines in their respective countries or regions reflect actual practice in the clinics without investigating within the clinics themselves [15]. Despite these limitations, this study does survey clinics from diverse countries (16 different countries) and the PIDS-IDSA, BTS, and WHO guidelines used for comparison are widely accepted and used in their respective areas.

It is difficult to establish a uniform definition and approach to the treatment of paediatric CAP in Europe due to the absence of a paediatric CAP severity score, the difficulty of identifying the aetiology, and differences in antibiotic resistance rates. Information concerning the changes in CAP epidemiology following the introduction of new vaccines against respiratory pathogens is also lacking [27]. Taking into account these difficulties, our survey managed to reveal the great variety of clinical and laboratory criteria used in the diagnosis and treatment of CAP. Heterogeneity between centers may reflect differences in epidemiology, aetiology, financial disparities and, in particular, vaccine usage, rate of disease and antibiotic resistance rates. While local treatment guidelines are likely to vary based on local resistance patterns, amoxicillin is suitable for most cases (especially outpatient) in most locations. Therefore, it can be concluded that although for diagnosis and treatment of CAP local variations in clinical criteria, laboratory tests, and antibiotic resistance rates may necessitate some differences in standard empirical antibiotic regimens, there is considerable scope for standardisation across European centres.

## Additional file

Additional file 1: Community-Acquired Pneumonia Paediatric Research Initiative (CAP-PRI) Questionnaire regarding diagnosis and treatment strategies of community acquired pneumonia distributed to paediatric infectious disease specialists at European medical centres. (DOC 50 kb)

## References

[1] Harris M, Clark J, Coote N, Fletcher P, Harnden A, McKean M, British Thoracic Society Standards of Care Committee (2011). British Thoracic Society guidelines for the management of community acquired pneumonia in children: update 2011. Thorax.

[2] Heath PT (2000). Epidemiology and bacteriology of bacterial pneumonias. Paediatr Respir Rev.

[3] Principi N, Esposito S (2011). Management of severe community-acquired pneumonia of children in developing and developed countries. Thorax.

[4] Ayieko P, English M (2007). Case management of childhood pneumonia in developing countries. Pediatr Infect Dis J.

[5] Grossman LK, Caplan SE (1988). Clinical, laboratory, and radiological information in the diagnosis of pneumonia in children. Ann Emerg Med.

[6] Bachur R, Perry H, Harper MB (1999). Occult pneumonias: empiric chest radiographs in febrile children with leukocytosis. Ann Emerg Med.

[7] Lynch T, Platt R, Gouin S, Larson C, Patenaude Y. Can we predict which children with clinically suspected pneumonia will have the presence of focal infiltrates on chest radiographs? Pediatrics. 2004;113:e186–9. PMID:14993575 http://pediatrics.aappublications.org/content/113/3/e186.short. 10.1542/peds.113.3.e186 14993575

[8] McIntosh K (2002). Community-acquired pneumonia in children. N Engl J Med.

[9] Juvén T, Mertsola J, Waris M, Leinonen M, Meurman O, Roivainen M (2000). Etiology of community-acquired pneumonia in 254 hospitalized children. Pediatr Infect Dis J.

[10] Rudan I, O’Brien KL, Nair H, Liu L, Theodoratou E, Qazi S, Child Health Epidemiology Reference Group (CHERG) (2013). Epidemiology and etiology of childhood pneumonia in 2010: estimates of incidence, severe morbidity, mortality, underlying risk factors and causative pathogens for 192 countries. J Glob Health.

[11] Haverkate M, D’Ancona F, Giambi C, Johansen K, Lopalco PL, Cozza V, VENICE project gatekeepers and contact points (2012). Mandatory and recommended vaccination in the EU, Iceland and Norway: results of the VENICE 2010 survey on the ways of implementing national vaccination programmes. Euro Surveill.

[12] ECDC Antimicrobial Resistance and Healthcare-Associated Infections Programme (2009). Antibiotic resistance in Europe: the challenges ahead. Euro Surveill.

[13] Bradley JS, Byington CL, Shah SS, Alverson B, Carter ER, Harrison C, Pediatric Infectious Diseases Society and the Infectious Diseases Society of America (2011). The management of community-acquired pneumonia in infants and children older than 3 months of age: clinical practice guidelines by the Pediatric Infectious Diseases Society and the Infectious Diseases Society of America. Clin Infect Dis.

[14] WHO. Pocket Book of hospital care for children. Guidelines for the management of common illnesses with limited resources. Second edition. Geneva: World Health Organization; 2013. p. 80–91. 24006557

[15] Ross RK, Hersh AL, Kronman MP, Newland JG, Metjian TA, Localio AR (2014). Impact of Infectious Diseases Society of America/Pediatric Infectious Diseases Society guidelines on treatment of community-acquired pneumonia in hospitalized children. Clin Infect Dis.

[16] Jain S, Williams DJ, Arnold SR, Ampofo K, Bramley AM, Reed C, CDC EPIC Study Team (2015). Community-acquired pneumonia requiring hospitalization among U.S. children. N Engl J Med.

[17] Thorburn K, Harigopal S, Reddy V, Taylor N, van Saene HK (2006). High incidence of pulmonary bacterial co-infection in children with severe respiratory syncytial virus (RSV) bronchiolitis. Thorax.

[18] Greenberg D, Givon-Lavi N, Sadaka Y, Ben-Shimol S, Bar-Ziv J, Dagan R (2014). Short-course antibiotic treatment for community-acquired alveolar pneumonia in ambulatory children: a double-blind, randomized, placebo-controlled trial. Pediatr Infect Dis J.

[19] O’Brien KL, Wolfson LJ, Watt JP, Henkle E, Deloria-Knoll M, McCall N, et al. Burden of disease caused by *Streptococcus* pneumoniae in children younger than 5 years: global estimates. Lancet. 2009;374:893–902. PMID:19748398, http://dx.doi.org/10.1016/S0140-6736(09)61204-6. 10.1016/S0140-6736(09)61204-6 19748398

[20] Watt JP, Wolfson LJ, O’Brien KL, Henkle E, Deloria-Knoll M, McCall N, et al. Burden of disease caused by Haemophilus influenzae type b in children younger than 5 years: global estimates. Lancet. 2009;374:903–11. PMID:19748399, http://dx.doi.org/10.1016/S0140-6456736(09)61203-4. 10.1016/S0140-6736(09)61203-4 19748399

[21] Koshy E, Murray J, Bottle A, Sharland M, Saxena S (2010). Impact of the seven-valent pneumococcal conjugate vaccination (PCV7) programme on childhood hospital admissions for bacterial pneumonia and empyema in England: national time-trends study, 1997-2008. Thorax.

[22] Kyaw MH, Lynfield R, Schaffner W, Craig AS, Hadler J, Reingold A, Active Bacterial Core Surveillance of the Emerging Infections Program Network (2006). Effect of introduction of the pneumococcal conjugate vaccine on drug-resistant Streptococcus pneumoniae. N Engl J Med.

[23] WHO Recommendations for routine immunization. World Health Organization; 2014. Available via WHO. http://www.who.int/immunization/policy/immunization_tables/en/. Accessed March 2015.

[24] Dagan R, Klugman KP. Impact of conjugate pneumococcal vaccines on antibiotic resistance. Lancet Infect Dis. 2008;8:785–95. PMID:19022193, http://dx.doi.org/10.1016/S1473-3099(08)70281-0. 10.1016/S1473-3099(08)70281-0 19022193

[25] Richter SS, Heilmann KP, Dohrn CL, Riahi F, Beekmann SE, Doern GV (2009). Changing epidemiology of antimicrobial-resistant Streptococcus pneumoniae in the United States, 2004-2005. Clin Infect Dis.

[26] ECDC. Antimicrobial resistance interactive database (EARS-Net). European Centre for Disease Prevention and Control; 2014. Available via ECDC. http://www.ecdc.europa.eu/en/healthtopics/antimicrobial_resistance/database/Pages/database.aspx. Accessed 30 March 2015.

[27] Esposito S, Cohen R, Domingo JD, Pecurariu OF, Greenberg D, Heininger U (2012). Antibiotic therapy for pediatric community-acquired pneumonia: do we know when, what and for how long to treat?. Pediatr Infect Dis J.

