# Supplementary material for: Comparison between diagnosis and treatment of community-acquired pneumonia in children in various medical centres across Europe with the United States, United Kingdom and the World Health Organization guidelines
Source: Pneumonia (Nathan). 2016 May 2;8:5. doi: 10.1186/s41479-016-0005-y (PMC5469201; doi:10.1186/s41479-016-0005-y)
Supplement: Additional file 1: — Community-Acquired Pneumonia Paediatric Research Initiative (CAP-PRI) Questionnaire regarding diagnosis and treatment strategies of community acquired pneumonia distributed to paediatric infectious disease specialists at European medical centres. (DOC 50 kb) [file 41479_2016_5_MOESM1_ESM.doc]

1. Please indicate the country you are working in: ...................................
2. Your main job is based in:

- outpatient clinic;
- emergency room;
- hospital ward;
- health care administration.

1. Do you use guidelines for community acquired pneumonia in children?

- Yes
- No

1. What kind of guidelines do you use?

- WHO;
- nationally accepted guidelines;
- approved only in your own hospital;
- Others (please specify).............................................

1. What criteria do you use for diagnosing community acquired pneumonia in children?

- cough;
- tachypnea;
- fever;
- intercostal, subcostal or suprasternal retractions;
- nasal flaring;
- crackles;
- decreased breath sounds;
- hypoxemia (oxygen saturation <95%);
- dehydration;
- abdominal pain;
- finding of consolidation in chest X-ray.

1. When chest X-ray is indicated in diagnosing pneumonia?

- never;
- routinely when pneumonia is suspected;
- only when clinical findings are ambiguous;
- hospitalization is required;
- persistent clinical symptoms are present despite AB treatment;
- complication of pneumonia is suspected;
- child < 5 years of age with fever of unknown origin.

1. Do you perform follow-up chest radiography:

- Yes
- No (please, go to question No. 10).

1. When do you perform follow-up chest radiography?

- after lobar collapse;
- apparent round pneumonia;
- for pleural effusion who required drainage;
- for continuing clinical symptoms;
- for lung abscess.

1. What laboratory investigations do you routinely perform in case of pneumonia who requires hospitalization:

- CBC (complete blood count);
- CRP (C-reactive protein);
- ESR (erythrocyte sedimentation rate);
- PCT (procalcitonin);
- serum electrolytes;
- blood culture;
- nasopharyngeal culture;
- sputum culture;
- rapid diagnostic tests (RSV, Adenovirus, Human metapneumovirus etc.) ;
- serology.

1. Criteria for hospitalisation:

- age <6 month;
- hypoxemia (oxygen saturation <92%, cyanosis);
- moderate to severe respiratory distress (respiratory rate >50 breaths/min in older children and >70 breaths/min in infants; difficulty breathing; intermittent apnoea, grunting);
- dehydration; inability to feed;
- underlying conditions (immunocompromised host, cardiopulmonary disease etc.) ;
- have failed outpatient antibiotic treatment;
- Family not able to provide appropriate observation or supervision.

1. What is the drug of choice for the Outpatient treatment of community-acquired pneumonia in children?

First line:

- amoxicillin;
- co-amoxiclav;
- cefuroxime;
- erythromycin;
- clarithromycin;
- azithromycin;
- Others (please specify).............................................

Second line:

- amoxicillin;
- co-amoxiclav;
- cefuroxime;
- erythromycin;
- clarithromycin;
- azithromycin;
- Others (please specify).............................................

1. Is the treatment choice is age dependent?

- No;
- Yes.

If yes, what is the treatment of choice:

<1-2months:

- - amoxicillin;
  - co-amoxiclav;
  - cefuroxime;
  - erythromycin;
  - clarithromycin;
  - azithromycin;
- Others (please specify).............................................

2 months to 2 years:

- amoxicillin;
- co-amoxiclav;
- cefuroxime;
- erythromycin;
- clarithromycin;
- azithromycin;
- Others (please specify).............................................

2-5 years:

- amoxicillin;
- co-amoxiclav;
- cefuroxime;
- erythromycin;
- clarithromycin;
- azithromycin;
- Others (please specify).............................................

>5 years

- amoxicillin;
- co-amoxiclav;
- cefuroxime;
- erythromycin;
- clarithromycin;
- azithromycin;
- Others (please specify).............................................

1. What is the drug of choice for treatment of community-acquired pneumonia in hospital in children?

First line:

- amoxicillin oral or IV;
- ampicillin oral or IV;
- co-amoxiclav oral or IV;
- benzyl pencillin; IV;
- cefuroxime oral or IV;
- cefotaxime IV;
- erythromycin oral or IV;
- clarithromycin; oral;
- azithromycin oral;
- Others (please specify).............................................

Second line:

- amoxicillin oral or IV;
- ampicillin oral or IV;
- co-amoxiclav oral or IV;
- benzyl pencillin; IV;
- cefuroxime oral or IV;
- cefotaxime IV;
- erythromycin oral or IV;
- clarithromycin; oral;
- azithromycin oral;
- Others (please specify).............................................

1. Is the treatment choice is age dependent?

- Yes;
- No.

If yes, what is the treatment of choice:

<1-2months:

- amoxicillin oral or IV;
- ampicillin oral or IV;
- co-amoxiclav oral or IV;
- benzyl pencillin; IV;
- cefuroxime oral or IV;
- cefotaxime IV;
- erythromycin oral or IV;
- clarithromycin; oral;
- azithromycin oral;
- Others (please specify).............................................

2 months to 2 years:

- amoxicillin oral or IV;
- ampicillin oral or IV;
- co-amoxiclav oral or IV;
- benzyl pencillin; IV;
- cefuroxime oral or IV;
- cefotaxime IV;
- erythromycin oral or IV;
- clarithromycin; oral;
- azithromycin oral;
- Others (please specify).............................................

2-5 years:

- amoxicillin oral or IV;
- ampicillin oral or IV;
- co-amoxiclav oral or IV;
- benzyl pencillin; IV;
- cefuroxime oral or IV;
- cefotaxime IV;
- erythromycin oral or IV;
- clarithromycin; oral;
- azithromycin oral;
- Others (please specify).............................................

>5 years:

- amoxicillin oral or IV;
- ampicillin oral or IV;
- co-amoxiclav oral or IV;
- benzyl pencillin; IV;
- cefuroxime oral or IV;
- cefotaxime IV;
- erythromycin oral or IV;
- clarithromycin; oral;
- azithromycin oral;
- Others (please specify).............................................
